# Testing a Mobile App for Participatory Research to Identify Teen-Targeted Food Marketing: Mixed Methods Study

**DOI:** 10.2196/35886

**Published:** 2022-05-03

**Authors:** Emily Truman, Charlene Elliott

**Affiliations:** 1 Department of Communication, Media and Film University of Calgary Calgary, AB Canada

**Keywords:** mHealth, mobile app, teenager, adolescent, monitoring, participatory research, feasibility, usability, food marketing, food advertising

## Abstract

**Background:**

Mobile apps are not only effective tools for promoting health to teenagers but are also useful for engaging teenagers in participatory research on factors that influence their health. Given the impact of food marketing messages on teenagers’ food attitudes and consumption choices, it is important to develop effective methods for capturing the food advertisements targeted at this population to assess their content.

**Objective:**

The aim of this study was to test the feasibility and usability of a mobile app, “GrabFM!” (“Grab Food Marketing!”), designed for teenagers to facilitate monitoring of self-identified targeted food marketing messaging.

**Methods:**

A mixed methods approach, including quantitative user response rates and qualitative focus group discussion feedback, was used in the evaluation process.

**Results:**

A total of 62 teenagers (ages 13-17) completed GrabFM! app pilot testing over a 7-day data collection period. Teenagers submitted a total of 339 examples of food marketing, suggesting high feasibility for the app. Participants also took part in focus group discussions about their experience, providing positive feedback on usability, including ease of use and design aesthetic appeal.

**Conclusions:**

The GrabFM! app had high feasibility and usability, suggesting its efficacy in capturing accurate data relevant to the teenage population’s experience with food marketing messaging.

## Introduction

Current research around mobile health (mHealth) and teenage populations focuses on health promotion via behavioral change interventions [1-6]. This includes studies employing the use of mobile technology to allow for self-monitoring of diet and exercise [7,8]. Indeed, mobile technology offers unique opportunities to access and collect data from the everyday lives of teenagers on topics that influence their health. Specifically, in light of growing evidence of the impact of food marketing messages on teenagers’ food preferences, attitudes, and consumption [9,10], it is important to learn more from this group about the food messaging they see and engage with.

To date, research that partners with teenagers to document the food marketing messages that they encounter has not used mobile app technology to empower teenagers to engage in self-reporting. Such participatory research instead uses body cams [11-14] and eyeglass cameras [15] to capture food marketing messaging that teens may be exposed to, but not necessarily *notice* or *engage* with (ie, identify as relevant). Using an mHealth approach, the project detailed in this article uses an evidence-based mobile app called “GrabFM!” (“Grab Food Marketing!”), which was designed for teenagers to facilitate self-reporting of targeted food marketing messages. This project thus addresses gaps in current participatory research on food advertising’s reach and persuasive content with respect to teenagers. This study further adds to knowledge about the mHealth elements (ie, mobile app user experience) and participatory methods (ie, data collection procedures) that facilitate the monitoring of health-related data for teenagers.

Reviews of existing literature show that mobile apps are an effective method for reaching teenagers when it comes to health promotion [16], and specifically for self-monitoring of health-related behaviors [17]. This is facilitated by increasing levels of smartphone accessibility among teenagers, and their familiarity and ease with new technologies [16,18]. The acceptability of smartphone use in different social settings for teenagers also contributes to their comfort level in their use for self-monitoring [7].

Current literature on the development of teen-oriented mHealth apps offers important insights into their assessment. A recent systematic review on the quality of mHealth apps for teenagers identified common rating criteria, including ease of use, visual appeal, interactivity, and degree of customizability [16]. Such criteria are commonly explored under the umbrella concepts of *feasibility* (ie, suitability to perform the intended tasks) and *usability* (ie, quality of user experience) [7,18]. Both feasibility and usability are directly relevant to the current study, in which a teen-oriented evidence-based mobile app was created to facilitate the self-identification of food marketing messaging to teenagers. Following an mHealth approach to evaluation, the aim of this study was to explore the app’s feasibility and usability for identifying teen-identified targeted food marketing messages. More broadly, this study contributes to gaps in knowledge around the persuasive power (ie, specific techniques or strategies used to persuade) and platforms of exposure of teen-targeted food marketing. Findings on the app’s feasibility/usability are important for both researchers engaging in mHealth behavioral interventions, as well as those exploring the use of mobile apps for participatory health-related research.

## Methods

### App Design Process

**Figure 1:**
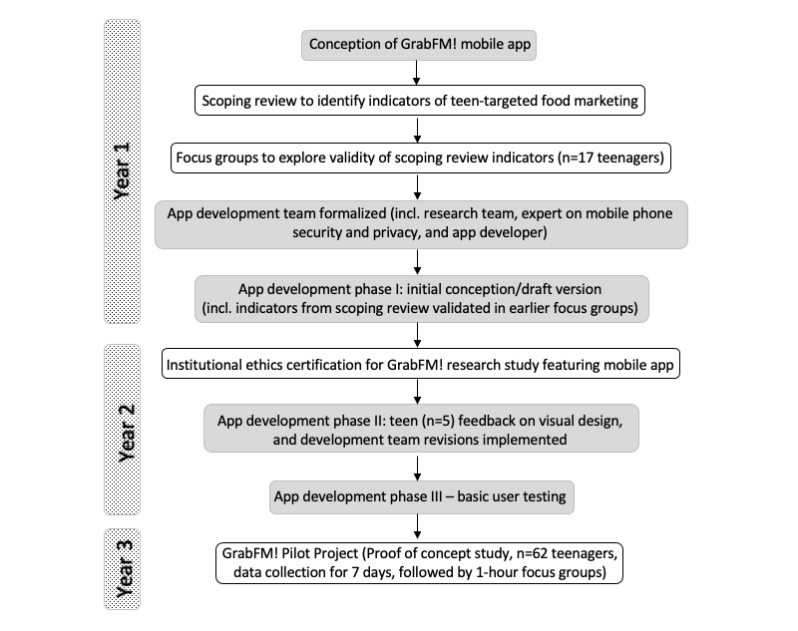
Flow chart of GrabFM! mobile app development.

The mobile app was developed iteratively with app developers (alongside an expert in app privacy and security) beginning in 2018 over a period of 3 years. Figure 1 outlines the basic steps involved in project development. The goal was to create a mobile app user experience that would allow teenagers (ages 13-17) to easily capture examples of food and beverage marketing (both online and in their physical environments using screenshots/photos and to tag those images with identifying information. The app content was informed by a scoping review of existing literature on identifying teen-targeted food marketing, highlighting relevant platforms and indicators (ie, marketing techniques) [19], and was assessed for appropriateness by a group of teenagers (n=17) in focus group discussions. Additionally, a small group of teenagers (n=5) provided feedback on the app’s visual design (ie, color, imagery) during the development process. App design elements include: a detailed tutorial (that launches automatically upon first opening the app), main interface for data submission (ie, upload screenshot/access camera functions, text fields, and preset lists of options for entering identifying metadata), an image library (with ability to select “favorite images”), and daily push notifications once a day for 7 days (beginning after first use of the app). The app was developed in both iOS and Android versions to accommodate a variety of devices.

### Ethics Approval

This study was approved by the University of Calgary Conjoint Faculties Research Ethics Board (REB19-0020).

### Sample and Study Procedure

A mixed methods approach was used, including participatory data collection and focus group discussions. Teenagers between the ages of 13 and 17 were recruited between January and May 2021 from schools (via school boards and research team member networks with principals and teachers), community groups, and sports teams. Principals, teachers, and other group leaders were contacted via email by the study team and invited to participate with their group. Given the low-risk nature of the study and the age of the participants, participants provided their own consent (with additional parental consent where required by schools). Once registered (through a secure website), participants were provided with a unique user code to gain access to the app after download (to their own device) from Apple App Store or Google Play Store. Participants then used the app for a 7-day period to submit self-identified examples of teen-targeted food marketing. Data submission involved the following steps: upload screenshot or take a photo of a food marketing example, and then input (1) the brand, (2) food type (independent text fields), (3) platform (ie, communication channel) where the example was found (selected from a preset list of 16 options, including “other” text field to add additional platforms), and (4) content “tags” indicating the teen-targeted marketing techniques used (selected from a preset list of 10 options, including “other” text field to add additional tags) [20].

Following the 7-day data collection period, the teenagers took part in focus group discussions to provide feedback on app usability (ie, likes and dislikes regarding functionality, appropriateness of evidence-based content, and barriers and facilitators to user engagement). Focus groups were conducted online due to restrictions around COVID-19 requiring limited in-person contact, using a semistructured moderator’s guide to facilitate discussion. Three researchers facilitated the process (Zoom meeting host/tech facilitator, moderator, and note-taker). Focus groups were recorded in Zoom, transcribed verbatim, and analyzed for themes by two researchers using Nvivo 12 software.

### Analysis

App feasibility was defined as the suitability of the app to perform the required tasks (ie, to engage and sustain participant engagement over the use period) [21]. This was measured quantitatively via response rates (as tracked through backend data), which were used for descriptive statistics [22]. App usability was defined as perceived ease of use and enjoyment of use [21,23]. Usability was examined through qualitative data from focus group discussions and presented in a descriptive summary [23]. Additionally, a qualitative approach was used to allow the teenagers to provide more in-depth responses regarding their experiences using the app for self-initiated data collection [23]. A thematic analysis was performed on the focus group discussion data to identify emerging themes.

## Results

### Participant Characteristics

Sixty-two teenagers used the app for a 7-day period between January and May 2021 (in small groups, with rolling time frames). Five participants were excluded from the sample due to incomplete submissions (ie, missing indicators/tags or images), leaving a sample size for app use of 57 teenagers. Teenagers also took part in focus group discussions upon completing the 7-day data collection period using the app. Seven 1-hour mixed-gender discussion groups were conducted between January and May 2021. A total of 47 teenagers provided feedback on their experience using the GrabFM! app in the discussion groups (note that the sample size is smaller for the focus group discussions, as some of the participants from the app data collection phase study were unavailable to participate in the focus groups). Table 1 provides a breakdown of participant demographics in terms of gender and age.

**Table 1:** Participant demographics.

Characteristic		App testing group, n	Focus groups, n
**Gender**			
	Girl	39	30
	Boy	17	17
	Gender nonconforming	1	0
**Age (years)**			
	13	23	15
	14	18	20
	15	9	9
	16	5	2
	17	2	1

### Feasibility

Overall, 339 self-identified examples of food marketing were submitted, suggesting high levels of feasibility for the app. Over the 7-day data collection period, an average of 6 ads per participant were submitted (range of 1-15 ads overall), with the exception of one outlier who submitted 47 ads.

### Usability

In the focus groups, teens reported high levels of usability. In general, they reported positively on overall ease of use (“smooth to run” [boy, age 14], “it was simple” [boy, age 14], “everything was labeled” [girl, age 15]), including easy image uploading (“easy to set and easy to upload” [girl, age 14], “loaded very fast” [boy, age 14]). They also provided positive feedback on the app’s esthetics (“The app itself looked nice” [boy, age 14], “I liked the color” [girl, age 14], “It’s very bright and vibrant” [girl, age 13]).

A few teens expressed difficulty in initially understanding how the app functioned (despite the fact that the tutorial was set up to launch automatically upon first opening of the app): “I didn’t know if I was supposed to take pictures of what I saw–had seen–in real life or what [else] I had to do before that. I couldn’t find the tutorial” (boy, age 14). Roughly one third of the participants also commented on the notifications feature with mixed impressions, including both positive (“I got daily notifications and they were helpful” [girl, age 14]) and critical (“I turned it on but I didn’t get any” [girl, age 13]) reviews. Finally, although most of the participants perceived the evidence-based list of platforms and indicators to be complete, a few additions were suggested for both platforms (Twitch [girl, age 13], Spotify [girl, age 16], Radio [girl, age 17], and Pinterest [girl, 14]) and indicators (ie, marketing techniques: trendy [girl, age 14; girl, age 14], sports [girl, age 13], and filters [girl, age 14]).

## Discussion

### Principal Findings

This pilot study suggests that the GrabFM! app is feasible given the sustained engagement by participants across the 7-day data collection period. Overall, the app’s usability was also highly rated in terms of ease of use and appeal of aesthetics. Good usability was also reflected in the high rate of complete data submission by participants; only 8% (n=5) of users were eliminated for missing information. This was an important outcome, as one of the central goals in developing the GrabFM! mobile app as a research tool was to allow for effective in-field data capture. As noted in the mHealth monitoring literature, self-reporting in-field is preferable as it does not require user recall, which can be unreliable [18].

Thus, to mitigate data loss based on incomplete submissions, and in light of the focus group feedback on unclear functionality, the results of this study highlight the need for comprehensive onboarding instructions (an external tutorial reinforcing the in-app tutorial) to ensure that all participants understand the procedure for accurate data collection. Further, given the importance of accurate data capture, the mixed feedback from the focus groups on the notification feature suggests the need to shift from manual settings (originally designed to allow for choice of start date) to automatic settings (one push notification per day for 7 days once the app is opened). Additionally, it is beneficial to instruct participants to set a daily timer on their phones as a reminder for data collection [24]. This will serve as a reinforcement mechanism.

In the focus group discussions, teenagers were asked to provide their own recommendations to increase teen buy-in to app use. Most of the suggested facilitators for buy-in revolved around gamification features such as rewards/points/prizes, monetary value on ad submissions, achievements/badges to unlock levels, competing against peers, linking to social media networks (increasing likes/friends/followers), and daily goals. Such features underline the importance of engagement strategies and social feedback mechanisms for teenagers when it comes to self-monitoring activities as noted in previous studies [6,16,25].

While gamification features (ie, goals/targets as behavioral motivators) are not directly relevant to the GrabFM! project given the app’s primary purpose as a data collection tool, these teen-identified facilitators to user engagement are useful for researchers developing mHealth interventions to promote attitude and behavior changes in relation to food/diet for teenagers, about which research remains limited [2,4,26]. Differences in gamification features aside, the important common feature of mHealth monitoring apps (eg, for dietary intake or physical activity levels) and the GrabFM! data collection app is the goal of facilitating self-monitoring (in the form of digital diary keeping) to increase user awareness around particular types of health-related information [17]. Indeed, although not explicitly designed as an intervention tool, the GrabFM! app promotes increased awareness of targeted food messaging, suggesting the need to further explore its educational potential in line with more traditional mHealth apps.

### Strengths and Limitations

The GrabFM! smartphone app is an innovative mHealth tool that is evidence-based, its content having been derived from the literature on monitoring teen-targeted food marketing messages. Further, the design/user experience was developed iteratively in conjunction with the app developer expressly for the teenage user to facilitate accurate data collection, along with careful considerations of privacy and security. However, it is important to note that this is a proof-of-concept pilot study with a small sample; as such, the feasibility and usability of the GrabFM! app will be further tested in the full project rollout currently underway with a much larger group of teenagers. Additionally, it is important to note that the content of the food marketing data collected during this pilot study is outside of the scope of this paper, which has been analyzed previously [20].

### Conclusion

Mobile apps are not only an effective method for promoting health to teenagers but are also useful tools for engaging this population in participatory research around factors that influence their health. This study used an mHealth approach to monitoring health-related information using a smartphone app that positions teen participants as experts in the identification of relevant food marketing messages that target them. Both the feasibility and usability of the GrabFM! app were found to be high with teenagers, suggesting its efficacy in capturing accurate data relevant to the teenage population’s experience with food marketing. These findings set the stage for use of the GrabFM! app in a broader study to provide important insights into the reach and content of targeted food marketing to teenagers as revealed in their self-identified data.

## Abbreviations

GrabFM!: Grab Food Marketing app
mHealth: mobile health
